# Impact of frailty and ultrasonography-based sarcopenia on the development of postoperative complications in gastrointestinal cancer patients

**DOI:** 10.3906/sag-2012-242

**Published:** 2021-06-28

**Authors:** Gözde ŞENGÜL AYÇİÇEK, Timuçin EROL, Pelin ÜNSAL, Olgun DENİZ, Osman ABBASOĞLU, Meltem HALİL

**Affiliations:** 1 Division of Geriatrics, Department of Internal Medicine, Hacettepe University Faculty of Medicine, Ankara Turkey; 2 Department of General Surgery, Hacettepe University Faculty of Medicine, Ankara Turkey

**Keywords:** Frailty, gastrointestinal cancer, older adults, sarcopenia, ultrasonography

## Abstract

**Background/aim:**

Gastrointestinal (GI) system cancers are frequent among older adults and it is still difficult to predict which are at increased risk for postoperative complications. Frailty and sarcopenia are increasing problems of older population and may be associated with adverse outcomes. In this study we aimed to examine the effect of sarcopenia and frailty on postoperative complications in older patients undergoing surgery for GI cancers.

**Materials and methods:**

Forty-nine patients admitted to general surgery clinic with the diagnosis of gastrointestinal system cancers were included in this cross-sectional study. Frailty status was assessed using the Edmonton Frail Scale (EFS). Sarcopenia was defined due to the EWGSOP2 criteria and ultrasonography was used to evaluate muscle mass.

**Results:**

The median age of the patients was 70 (min-max: 65–87). Fourteen (28.6%) patients were found to be sarcopenic and 16 (32.7%) patients were frail, and 6 (37.5%) of these patients were also severe sarcopenic (p = 0.04). When the postoperative complications were assessed, time to oral intake, time to enough oral intake, length of hospital stay in the postoperative period were found to be longer in frail patients (p = 0.02, p = 0.03, p = 0.04 respectively). Postoperative complications were not different due to the sarcopenia.

**Conclusion:**

Frailty, but not sarcopenia was associated with adverse outcomes in older adults undergoing GI cancer surgery. Comprehensive geriatric assessment before surgical intervention may help to identify patients who are at risk.

## 1. Introduction 

Gastrointestinal (GI) system cancers are common and responsible for the one third of cancer-related deaths [1]. Surgical resection is the main treatment of these patients. It is still difficult to predict which patients are at increased risk for postoperative complications. Several studies have pointed out that older patients carry potential risks in surgery and may encounter more adverse postoperative outcomes compared to younger patients [2].

Frailty is a vulnerable state that is characterized with an insufficient response to a stress condition and improvement in homeostasis following stress. In frailty risk of adverse outcomes such as disability, delirium and falls were increased [3]. Frailty was found to be one of the strong prognostic factors of survival in colorectal cancer patients apart from the tumor characteristics [4]. 

Sarcopenia, is characterized by progressive loss of muscle mass and muscle function in the older adults. This geriatric syndrome has been found to be related to adverse clinical outcomes including high risk of hospitalizations, falls, functional impairment, fractures, and mortality [5]. Although handgrip strength and gait speed are commonly utilized in the assessment of muscle strength and physical performance, the gold standard for the evaluation of muscle mass is still controversial. Among the imaging studies, muscle ultrasound (US) seems promising as previous studies have revealed that it is a reliable and valid technique to assess muscle mass and is superior to other techniques with its noninvasive, portable, radiation-free, easy, and repeatable properties [68]. Sarcopenia prevalence is high in GI cancer patients and related with adverse outcomes including poor survival, postoperative infection, and chemotherapy toxicity [9,10].

There is an overlap between frailty and sarcopenia. Fried et al. defined the physical frailty phenotype with the existence of exhaustion, low grip strength and low gait speed, self-reported low physical activity, and weight loss [11]. Older adults who could most benefit from a Comprehensive Geriatric Assessment (CGA) could be identified with the frailty assessment [12]. Several screening tools have been invented to assess frailty in clinical practice, some of them only assess physical frailty while others investigate multiple domains [13]. The Edmonton Frail Scale (EFS) has been validated by Rolfson et al. to assess multi-dimensional presentations of frailty in older adults [14]. The test has also been validated in the Turkish population [15].

In this study, it is purposed to examine the impact of frailty and sarcopenia on postoperative complications in older patients undergoing surgery for GI cancers.

## 2. Materials and methods

Forty-nine patients admitted to general surgery clinic with the diagnosis of gastrointestinal system cancers were enrolled in this cross-sectional study. Their medical history was taken and all subjects were underwent physical examination. Comorbid illnesses and current medications were noted. Patients with prosthesis, acute infection, severe edema, acute cardiac diseases (decompensated congestive heart failure, recent myocardial infarction/stroke), pacemakers, who cannot cooperate, outpatient surgery patients, emergency surgery patients, patients operated under local anesthesia, and terminal cancer patients who underwent palliative surgery were excluded.

### 2.1. Comprehensive geriatric assessment protocol and anthropometric measurements 

All patients underwent comprehensive geriatric assessment (CGA). Nutritional status was evaluated with Mini Nutritional Assessment–short form [16]. Activities of daily living and instrumental activities of daily living were assessed with Katz and Lawton–Brody tests, respectively [17–20]. Mini Mental State examination was used for cognitive functions [21], the mood assessment was performed by the Yesavage Depression Scale [22]. 

Frailty status was assessed using Edmonton Frail Scale (EFS). The EFS evaluates 9 parameters of frailty such as general health status, cognition, medication usage, functional independence, nutrition, social support, mood, functional performance, and continence. The total score of the scale changes from 0 to 17. The participants were classified to the EFS score as: no frailty (<5), apparently vulnerable (5–6), mild frailty (7–8), moderate frailty (9–10), and severe frailty (≥11) respectively [14].

Weight, height, upper-mid arm, waist, hip, calf, circumferences were measured and body mass index (BMI, kg/m2) was calculated at hospital admission.

### 2.2. Muscle strength and physical performance measurements 

Sarcopenia was defined due to the 2018 EWGSOP2 criteria [8]. Sarcopenia was defined as probable in the presence of low muscle strength. The diagnosis of sarcopenia was confirmed with the addition of low muscle quality or quantity to low muscle strength. Severe sarcopenia definition was performed with low muscle strength, low muscle quantity or quality and low physical performance. Grip-D, grip strength dynamometer (Takei, HaB International Ltd., Warwickshire, UK) was used to measure muscle strength from dominant hand. After 10 s intervals measurements were repeated for three times and maximum hand grip strength value was recorded. For males 27 kg and for females 16 kg were used as the cut-off thresholds [8]. Gait speed measurement have been performed to assess physical performance and a gait speed ≤ 0.8 m/s for 4 m was accepted as reduced physical performance and walking disability [8].

### 2.3. Muscle mass measurement 

All the measurements were performed by the same investigator. Bodystat Quadscan 4000 device (FL, USA) was used for bioelectrical impedance (BIA) measurement from the right side of the body in supine position. Electrodes placed on the dorsal side of the wrist (between the distal prominences of the radius and ulna) and the dorsal side of the ankle (between the medial and lateral malleoli) joints. Fat free mass index (FFMI) values were recorded. By using the following formula: SMI (kg) = 0.566 ∗ FFMI, skeletal muscle mass index (SMI) was calculated [23] and was used to estimate muscle mass. Cut off points for skeletal muscle mass index was validated as < 9.2 kg/m2 for men and < 7.4 kg/m2 for women in our population [23].

### 2.4. Ultrasonographic evaluations

A linear probe with 5–12 MHz (LOGİQ 200 PRO, General Electrics Medical Systems, Ultrasmed, Neuss, Germany) was used to perform US by the same physician, who was blinded to the study data and who had at least 10 years of experience in the issue of musculoskeletal US. Measurements were performed from 6 different types of muscle, rectus abdominis (RA), internal abdominal oblique (IO), external abdominal oblique (EO), transversus abdominis (TA), rectus femoris (RF), and gastrocnemius medialis (GM). Measurements were performed in direction of the recommendations of the European Union Geriatric Medicine Society Sarcopenia Special Interest Group [24] and to the recent literature [25]. Minimal pressure was applied by the US probe on the right side of the body at the selected sites during all measurements. To control the effect of respiration abdominal muscles (RA, IO, EO, and TA) were measured at the end of a normal exhalation [26]. Cross-sectional area (CSA) was defined as the area of the cross section of a muscle perpendicular to its longitudinal axis. 

### 2.5. Postoperative evaluation

Postoperative complications developed after surgery were classified as infectious and noninfectious. Length of hospital and intensive care unit stay, reoperation requirement, time to oral intake (TTOI), time to enough oral intake (TTEOI), and the development anastomotic leakage were also recorded.

### 2.6. Statistical analysis

The Statistical Package for Social Sciences (SPSS) for windows v:15.0 (IBM Corp., Armonk, NY, USA) software was used to perform statistical analysis. To determine whether the variables had normally distributed or not, histograms and Shapiro–Wilk test were performed. Categorical variables were given as frequencies (n) and percentage (%). Continuous numerical parameters were compared between two groups by using Student’s t or Mann–Whitney U tests. Comparison of categorical parameters was performed with Chi-square test. The relationships were assessed with Pearson’s correlation analysis test for normally distributed variables and with Spearman’s correlation analysis for not normally distributed variables. A value of p < 0.05 was accepted as significant. 

## 3. Results

A total of 49 patients were enrolled in the study. The median age of the patients was 70 (range: 65–87) and 49% (24) of the patients were female. Fourteen (28.6 %) patients were found to be sarcopenic. Female patients were more sarcopenic (54.2%) compared to males (p < 0.001). A total of 16 (32. 7%) patients were frail with EFS, and 6 (37.5%) of these patients were also severely sarcopenic (p = 0.04). Descriptive characteristics and results of CGA parameters are shown in Table 1. 

**Table 1:** Descriptive characteristics and CGA results of the patients.

	Frail(n = 16)	Non frail(n = 33)	p	Sarcopenic(n = 14)	Non sarcopenic(n = 35)	p
Age	74.1 ± 5.9	70.0 ± 4.6	0.02	71.2 ± 4.6	71.4 ± 5.7	0.892
Sex (female)	9(56.2%)	15(45.4%)	0.483	13(93%)	11(31.4%)	<0.001
BMI	25.2 ± 6.2	25.9 ± 4.4	0.683	23.3±5.0	26.7 ± 4.8	0.05
KATZ	5(2–6)	6(5–6)	<0.001	6(2–6)	6(3–6)	0.344
Lawton Brody	6(2–8)	8(5–8)	<0.001	8(2–8)	8(2–8)	0.103
MNA	10(5–13)	11(8–14)	0.006	9(5–14)	10(6–14)	0.128
MMSE	25(10–29)	28(20–30)	0.002	27(20–30)	28(10–30)	0.386
YDS	3(1–10)	2(0–5)	0.005	3(1–10)	2(0–5)	0.096

BMI: Body mass index, MNA: Mini nutritional assessment, MMSE: Minimental state examination, YDS: Yesavage depression scale.

Thirteen (26.5 %) patients had diabetes mellitus (DM) and the median HbA1C level was 7 (min: 4.6–max:11.7). After good diabetic control all patients underwent surgery. Twenty six (53.1%) patients had hypertension (HT) and 6 (12.2%) patients had coronary artery disease (CAD). Of the 49 patients, 45% underwent surgery for colon cancer, 24.5% for stomach cancer, 8.2% for esophagus cancer, 8.2% for rectum cancer, 6.1% for pancreas cancer, and 8% for other GI cancers. Only one patient received chemotherapy before the surgery.

All measured muscle thicknesses were thinner in frail patients compared to nonfrail patients, except for TA. Six different areas of muscle thicknesses were all lower in sarcopenic patients (Table 2).

**Table 2:** Relationship between muscle thickness, frailty, and sarcopenia.

	Frail(n = 16)	Non frail(n = 33)	p	Sarcopenic(n = 14)	Non sarcopenic(n = 35)	p
GM	11.1 ± 1.8	12.7 ±2.0	0.009	10.6 (1.5)	12.9 (1.9)	<0.001
RF	9.35 ± 2.6	11.93 ± 3.01	0.004	9.4 (2.8)	11.7 (3.0)	0.019
RF CSA	3.4 ± 1.2	4.5 ± 1.6	0.02	3.2 (1.1)	4.6 (1.6)	0.008
RA	3.03 ± 1.7	6.8 ± 1.36	0.04	5.5 (1.5)	7 (1.3)	0.002
EO	3.05 (2-5)	3.9 (2.2-6.7)	0.013	3.5 (2–4.1)	3.9 (2.2–6.7)	0.02
IO	4.5 ± 1.35	5.4 ± 1.37	0.024	5.5 (1.3)	4.2 (1.2)	0.002
TA	3.2 ± 0.92	3.6 ± 0.81	0.101	3.0 (0.9)	3.7 (0.8)	0.007

GM: Gastrocnemius medialis, RF: Rectus femoris, RA: Rectus abdominis, EO: External abdominal oblique, IO: Internal abdominal oblique, TA: Transversus abdominis.

When the postoperative complications were assessed, TTOI, TTEOI, length of hospital stay (LOS) after surgery found to be longer in frail patients (p = 0.02, p = 0.03, p = 0.04 respectively). Postoperative complications were not different due to sarcopenia (Table 3).

**Table 3:** Postoperative complications due to frailty and sarcopenia.

	Frail(n = 16)	Non frail(n = 33)	p	Sarcopenic(n = 14)	Non sarcopenic n = 35)	p
Wound infection	5(31%)	12(36%)	>0.05	4(28%)	15(42%)	>0.05
Noninfectious complications	1(6.6%)	3(10.3%)	>0.05	1(7%)	3(10%)	>0.05
Reoperation requirement	1(6.6%)	2(6.8%)	>0.05	1(7%)	2(6%)	>0.05
Development anastomotic leakage	1(6.6%)	2(6.8%)	>0.05	1(7%)	2(6%)	>0.05
Intensive care unit stay	7(43%)	9(27%)	>0.05	13 (39%)	3 (21%)	>0.05
TTOI	4(0–11)	2(0–5)	0.02	3.3 ± 1.4	3.9 ± 2.5	>0.05
TTEOI	5(0–16)	3(0–7)	0.03	4.5 ± 2.2	4.7 ± 3.1	>0.05
LOS	7(5–28)	6(0–15)	0.04	13(3-36)	12(3-32)	>0.05

TTOI: Time to oral intake, TTEOI: Time to enough oral intake, LOS: Length of hospital stay.

In the correlation analysis, time to oral intake (r = 0.315, p = 0.02), time to enough oral intake (r = 0.312, p = 0.03) and LOS (r = 0.303, p = 0.03) were positively correlated with frailty (Table 4).

**Table 4:** Results of the correlation analysis.

	GM	RF	RF CSA	RA	EO	IO	TA	TTOI	TTEOI	LOS
EFSrp	–0.3490.014	-0.3720.008	-0.3080.031	NS	-0.3570.012	NS	NS	0.3310.02	0.3500.014	0.3030.034
Sarcopeniarp	–0.502<0.001	-0.2920.04	-0.3770.008	-0.4090.004	-0.3320.020	-0.3890.006	-0.3600.011	NS	NS	NS

EFS: Edmonton frailty scale, GM: Gastrocnemius medialis, RF: Rectus femoris, RA: Rectus abdominis, EO: External abdominal oblique, IO: Internal abdominal oblique, TA: Transversus abdominis, TTOI: Time to oral intake, TTEOI: Time to enough oral intake, LOS: Length of hospital stay.

## 4. Discussion

In this study, we investigated the role of frailty and sarcopenia on predicting outcomes in older patients undergoing surgery for GI cancers. Our results revealed that, frailty but not sarcopenia was associated with adverse outcomes in this population, pointing out the importance of comprehensive geriatric assessment with multiple domains.

Previous studies revealed that the state of frailty and sarcopenia in the preoperative period were related to the development of adverse postoperative outcomes, including increased morbidity and mortality, and prolonged LOS [27]. Makary et al. concluded that preoperative frailty was related to an increased risk of postoperative complications. The investigators reported that frailty independently predicted longer length of hospital stay with moderate (44%–53%) or severe frailty (65%–89%) than nonfrail subjects [28]. In the present study, we have shown that, time to oral intake, time to enough oral intake, length of hospital stay in the postoperative period was longer in frail patients. Additionally, time of oral intake, time of enough oral intake, and LOS were negatively correlated with frailty. While the physical domain of frailty, known as sarcopenia, was not associated with postoperative outcomes, multi-dimensional assessment of frailty with EFS revealed that frailty was associated with adverse outcomes after surgery. Frailty assessment during comprehensive geriatric assessment could help to identify older patients who may benefit from perioperative rehabilitation program. The EFS is a multifactorial scale, which is easy and quick to administer and prior geriatric assessment is not required [14]. The tool was found to be reliable and valid compared to a geriatrician’s clinical impression of frailty [14,15]. 

The importance of sarcopenia in the prediction of outcome after gastrointestinal surgery has been shown previously in several studies [29,30]. There are many different techniques to evaluate muscle mass including biochemical parameters, anthropometric measurements, bioimpedance analysis, and radiological tools such as computed tomography and magnetic resonance imaging [6]. Among imaging methods, muscle US seems promising and superior to others with its noninvasive, portable, radiation-free, easy, and repeatable properties. In this study we couldn’t find any correlation between sarcopenia and postoperative complications. This may be due to the small number of participants. However, we have shown that, sarcopenia was higher in frail patients than nonfrail patients. Also, all muscle thicknesses were thinner in frail patients. When we consider the link between sarcopenia and frailty, the positive correlation between frailty and postoperative complications, may be due to loss of muscle mass.

The study had several limitations. The small sample size could be the first limitation that we could not find statistical significance between some parameters especially with sarcopenia. Further studies with higher number of patients may reveal more reliable results. Additionally, due to heterogeneity of population, cancer types were not big enough to make differential subgroup analysis. The study is in a cross-sectional design, postoperative long term follow up should be performed to show the progressive decline in physical performance in frail and sarcopenic patients. This study was conducted in a single center, which may limit the generalization of the results. 

For older patients undergoing gastrointestinal surgery for cancer, frailty should be assessed in a multidimensional manner for optimal management. Identifications of patients who are prone to development of complications may help to improve patient outcome. Further studies are needed with larger number of patients to clearly define the impact of frailty and sarcopenia on the development of postoperative complications.

## Informed consent

The Hacettepe University Ethical committee approved the study (GO 18/696-21). This research was carried out according to the guidelines of the Helsinki Declaration. All participants signed informed consent form.
